# Osteoporosis, Activities of Daily Living Skills, Quality of Life, and Dietary Adequacy of Congregate Meal Participants

**DOI:** 10.3390/geriatrics3020024

**Published:** 2018-05-10

**Authors:** Fatma G. Huffman, Joan A. Vaccaro, Gustavo G. Zarini, Edgar R. Vieira

**Affiliations:** 1 Department of Dietetics and Nutrition, Florida International University, Miami, FL 33199, USA; jvaccaro@fiu.edu (J.A.V.); gzarini@fiu.edu (G.G.Z.); 2 Department of Physical Therapy, Florida International University, Miami, FL 33199, USA; evieira@fiu.edu

**Keywords:** osteoporosis, dietary requirements, self-rated health, activities of daily living (ADL), congregate meals

## Abstract

Osteoporosis, a chronic disease that results in low bone mass with an increased risk of fragility fractures, is prevalent in older adults. Diet can prevent or lessen the severity of osteoporosis. The purpose of this cross-sectional study was to assess differences in diet, quality of life, self-rated health, and physical function between congregate meal participants with and without osteoporosis. Data were from telephone survey, 10th Annual National Survey of Older American Act Participants, a representative sample of congregate meal attendees across the United States. (*N* = 888). Osteoporosis was present in 20% of this population. Participants with, as compared to without, osteoporosis reported that their physical health limited moderate activities (31.5% vs. 18.9%, *p* = 0.026), stair climbing (32.2% vs. 22.8%, *p* = 0.032), and shopping (27.4 vs. 15.3, *p* = 0.018). More than half of the participants consumed less than the recommended servings of dairy, meat, grains, and fruits/vegetables regardless of osteoporosis status. Participants with osteoporosis had lower self-rated health and more physical limitations than people without osteoporosis. Although congregate meals are a way to improve nutritional intake, additional methods to improve nutrition (including education) may be of benefit, since undernutrition is a concern in this population.

## 1. Introduction

Older adults are the fastest growing segment of the population and they have the highest medical cost of all age groups in the United States [1]. Primary (risk reduction), secondary (early detection), and tertiary prevention (management to prevent complications) of chronic diseases is critical given the older adult population growth and the increase in life expectancy because these factors are concurrent with increased prevalence of chronic diseases and healthcare spending [2]. Approximately 38% of deaths in the United States are due to behaviors such as tobacco use, poor diet and low physical activity, and excessive alcohol use [3]. Despite the rising cost of healthcare, the low priority placed by the healthcare system on primary disease prevention has resulted in increased morbidity, mortality, and lower quality of life [2].

Osteoporosis is a degenerative disease of the skeletal mineral content resulting in low bone mass with increased risk of fragility fractures. Bone mineral density for persons with osteopenia (low bone density) is between 1 and 2.5 standard deviations below, and for persons with osteoporosis is ≥2.5 standard deviations below the mean density of young, non-Hispanic White adults; osteoporosis affects 24.8% of women and 5.6% of men aged 65 and older [4], and approximately half of adults aged 50 years and older in the United States suffer from either osteopenia or osteoporosis [5]. Osteoporosis presents a burden and diminishes quality of life similar to other chronic diseases, [6] is associated with significant medical costs, and represents a significant public health problem [7]. 

Diet is a critical and modifiable risk factor for osteoporosis; calcium and vitamin D and an overall healthy diet including adequate protein from meat, eggs, beans, nuts, tofu, and sufficient vitamins, and minerals from fruits and vegetables are essential for adequate bone mass [8,9,10]. An important aspect of diet quality is meeting the requirements for the major food groups: fruit and vegetable; grain; protein food (meat, eggs, beans, seeds, tofu); and dairy [11]. 

Other lifestyle factors associated with reduced risk of osteoporosis are tobacco and second-hand smoke cessation, reduced alcohol intake, and increased weight-bearing exercise [12]. Osteoporosis has no symptoms; therefore, preventive and screening measures are necessary to prevent osteoporotic fractures and the associated consequence of functional decline [9]. Nutrition education, physical activity and health screenings (including bone density screening) are available at many congregate meal sites; however, a small proportion of attendees use these services [13]. The purpose of this study was to assess differences in quality of life, self-rated health, diet and physical function between congregate meal participants with and without osteoporosis and to assess dietary differences by sex.

## 2. Materials and Methods 

### 2.1. Study Design, Ethics, and Population

This study is a cross-sectional analysis of data from the 2015 Tenth Annual National Survey of Older American Act Participants (NSOAAP). The data are available to the public. The research protocol was approved by the Office of Management and Budget (OMB); the OMB Control Number for the 2015 Tenth NSOAAP is: 0985-0023. All participants signed an informed consent form for public use of their de-identified data. A two-staged stratified selection of 312 out of 628 area agencies on aging (AAoA) method was conducted and described in the Agency for Community Living website [14]. Briefly, the first stage was the selection of the AAoA and the second stage was the selection of the services within the AAoA. Weighting of each service record was done separately. Base weights were computed by taking the inverse of the selection probability for each sampled client, adjusted for nonresponse, then trimming of the extreme weights. Finally, a post-stratification adjustment was made using available control totals [14]. Fay’s modified Balanced Repeated Replication method was used for computation of the sampling variances of survey estimates. Under the modified approach, the full-sample weights are adjusted or “perturbed” to define the required replicates, rather than taking one variance unit from each stratum [14]. 

The data for this study were extracted from Congregate Meals, which was one of the six services; other services included Home Delivered Meals, Homemaker Services, Transportation, the Family Caregiver Support Program, and Case Management. The 2015 NSOAAP Congregate Meals survey was conducted by telephone. Participants were asked, by trained interviewers, about their socio-demographics, foods consumed at the site and throughout the day, degree of satisfaction with meals and services that were received, medical health, emotional health, functionality, and social life. Sample weights and variance estimation were applied to account for non-response and to achieve a representative sample of congregate meal attendees across the United States. The population included 901 adults ages 60 years and older who completed the 2015 NSOAAP Congregate Meals survey. The final sample size was *N* = 888 which had data available for osteoporosis diagnosis (absent/present).

### 2.2. Study Variables

The dependent variable, osteoporosis, was determined by the participants recalling and furnishing an affirmative response to ‘having a medical doctor tell you that you had osteoporosis.’ The independent variables were activities of daily living skills, dietary adequacy (recommended daily servings from major food groups: meat/protein, dairy, grain, fruits and vegetables), quality of life, and race/ethnicity.

- Level of dietary adequacy was considered as servings per day: under, at, or over the dietary guidelines’ recommendations for older adults for meat/protein-group (eggs, tofu, beans, seeds, fish, and other meats), grains, dairy, total fruit and vegetable intake, and dessert. Dessert is not considered a major food group; albeit, it is considered as discretionary calories along with added sugars, solid fats, and alcohol. For persons eating from 1000 to 1600 calories, only 100 to 170 calories per day remain for discretionary calories [15]. This would be approximately a half-portion of dessert a day, not considering other discretionary calories.
- Dietary recommendations for adults >50 years of age from each food group are as follows: 3.5 and 4.0 servings of fruits and vegetables; eight and nine servings of grain; five and 5.5 servings from the protein group; for women and men, respectively; and, three servings of dairy for both sexes [11].
- Dietary information was collected by interviewers who were trained in interpreting participants’ responses to type and amount of food consumed by probing interview questions sufficient to collect these details. They were asked to consider the foods they usually eat in a day. They could answer independently or with the help of their caretaker. The specific food group was presented. The following is an example of the script: “Considering all the food {you eat/s/he eats} in a day, how many servings of fruit {do you/does NAME OF PARTICIPANT} usually eat? One serving of fruit is one piece of fruit; one-half cup chopped, cooked, or canned fruit; or three-fourths cup of juice”.
- Difficulty with activities of daily living (ADL) skills were assessed by an affirmative response to questions concerning dressing, shopping, cooking, cleaning, and other physical activities.
- Physical health interfering with physical function was assessed by the question: “during the past four weeks, how much of the time have you accomplished less than you would like as a result of your physical health?”. The categories were collapsed into: all or most of the time, some of the time, and little or none of the time. This question is part of the Medical Outcome Study, RAND 36-item Short Form Survey Instrument [16].
- Self-rated health (SRH) was assessed based on the question: “in the past 12 months how would you rate your health?” Responses were collapsed from five categories (excellent, very good, good, fair, or poor) to three (fair/poor, good, or very good/excellent). SRH has been validated against actual health in older adults, and it is an independent predictor of mortality [17,18,19]. This question is also part of the Medical Outcome Study, RAND 36-itm Short Form Survey Instrument [16].
- Race/ethnicity was constructed by coding the responses for Hispanic and subtracting Hispanics from other categories to create non-Hispanic Whites, non-Hispanic Blacks, Hispanics, and Others (including Asians, American Indians, and Pacific Islanders).
- Other variables included activities at the site (attending nutrition education, exercising at the site, and receiving a health screenings); self-reported diabetes; education level; sex; marital status; food security (having or not having enough money to buy food), and low income (income < $20,000 per year).

### 2.3. Statistical Analysis

Characteristics of the study population were determined by frequency analysis with the test of equal cell proportions and presented as percentages with 95th confidence intervals. Diet and ADL were cross-tabulated by osteoporosis status and the Pearson chi-square test was used for testing differences between groups. Dietary adequacy was compared by sex. Separate reduced and full logistic regression models were created for SRH and dietary adequacy as major independent variables with osteoporosis as the dependent variable. Full models included major sociodemographics (age, gender, and race/ethnicity) and the rest of the study variables. The reduced model was the most parsimonious model (goodness of fit, G statistic) that retained the hypothesized predicting independent variable and the major sociodemographics. Analyses were performed with the Statistical Package for the Social Sciences (SPSS) version 24 (IBM, New York, NY, USA, 2017), with the module for complex sample analysis. Sample weights applied to achieve a representative sample of the United States population attending congregate meals. The results were presented with and without the Bonferroni correction for multiple comparisons. 

## 3. Results

The general characteristics of the study population are shown in Table 1. There were twice as many females as males, and 60% of the participants were single/divorced/widowed. Approximately half of the participants lived below the poverty level, and 15% did not have enough money to buy food. Nearly half reported that their physical health interfered with their accomplishments most of the time and 20% had recalled being told they had osteoporosis. Frequency differences of marital status and osteoporosis in each sex were no longer significant after applying the Bonferroni correction.

Table 2 presents a cross-tabulation and the chi-square test of significance for ADL difficulties and sex differences with and without osteoporosis. For persons with osteoporosis, there was a greater proportion reporting ADL difficulties for shopping, visiting a doctor, or housework. These differences were no longer significant after applying the Bonferroni correction. Fewer adults with osteoporosis reported that their physical health interferes little or none with their accomplishments as compared to those without osteoporosis. This indicator remained significant after the Bonferroni correction (*p* < 0.008) was considered. Medical conditions, such as joint pain and arthritis, and comorbidities, such as diabetes and anemia, were also associated with physical function (data not shown) and may be confounders for physical function and osteoporosis. 

Table 3 displays dietary adequacy by sex. It is likely that men consumed more calories per day than women because they consumed significantly more grains and desserts with no significant differences in meat, dairy, and fruit and vegetable groups; however, absolute calories were not available. Servings per day from all dietary food groups were below the recommended amounts: meat/protein (women, 5; men, 5.5), dairy (3 for both sexes), grains (women, 8; men, 9), and total fruit and vegetables, exclusive of potato, (women, 3.5; men, 4.5) for all regardless of osteoporosis status. Altogether, less than 10% of the people surveyed met the recommendation for the protein/meat group and less than 15% met the recommendation for dairy. Nearly all participants reported eating fruits and vegetables at the congregate site; yet, less than 25% met the recommendation for four servings from the fruits and vegetables groups, combined. Only estimated calories from congregate meals and grain remained significant after the Bonferroni correction (*p* < 0.007) was applied.

The dietary recommendation for total fruits and vegetables does not include potatoes, which accounted for some calories. Total consumption of vegetables, including potatoes, for men and women was as follows: 9.5%, one serving; 45.9%, two servings; 27.0, three servings; 10.7%, four servings; and 4.5%, five servings, with 2.4% misclassified or missing (data not shown in Table 3). Even when considering servings of potatoes as part of the fruit and vegetable group, men and women did not meet the recommended servings per day for all the major food groups, suggesting the majority of congregate meal site participants are not getting enough total calories, protein, calcium, and other vitamins and minerals from their food. Considering diet and osteoporosis, an unadjusted model (dietary without sociodemographics) could not be fitted (model classification, *p* = 0.235), nor could a model by race and sex (model classification, *p* = 0.065). 

There were no significant differences in SRH between those with and without osteoporosis in the unadjusted model; however, after adjusting for age, sex, race/ethnicity, and food security, those with osteoporosis had twice the odds of reporting fair to poor SRH compared to those without osteoporosis (Table 4).

## 4. Discussion

The aim of the present study was to compare diet, physical function, and a quality of life measure of participants attending congregate meals across the United States with and without osteoporosis. Clinically-important findings of this study were that persons reporting recalling a medical doctor told them they had osteoporosis were associated with poorer self-rated health, physical function, and undernutrition. Another important finding was that there were sociodemographic differences in dietary adequacy in the combined population (with and without osteoporosis). 

Persons surveyed in this study had below the recommended servings from the meat and dairy groups placing them at risk for osteoporosis or the disease progression. The International Osteoporosis Foundation [10] recommends adequate dietary protein from dairy and lean red meat, poultry, fish, eggs, soy products, whole grains, nuts and seed is essential for maintaining muscle strength and bone mass. Bone health depends on adequate amounts of calcium (bioavailable in dairy), magnesium (found in nuts, seeds, grains, and green vegetables, vitamin A (found in red and yellow fruits and green vegetables), vitamin K (abundant in leafy-green vegetables), and zinc (found in red meats, whole grains, and legumes) [10]. In particular, participants consumed less than the recommended five servings of fruits and vegetables per day for vitamins, mineral, and fiber; less than the recommended minimum of dairy (three servings per day) for calcium, vitamin D, and protein; less than adequate protein from the meat group. These results suggest that adequate protein, calcium, vitamins, and minerals, essential for bone health, are not being attained from food consumption by congregate meal site attendees across the United States. 

Sociodemographic differences in diet adequacy were present in Florida congregate meal attendees. Thirty-one percent of older adults in six congregate meal sites in Northern Florida were at high nutrition risk with the majority being African-American and female [20]. In the current study, 80% of men and women are not meeting their requirements from the protein/meat and dairy groups; half of men and approximately 40% of women are not meeting requirements for the combined fruit and vegetable group; and 85% of men and 90% of women are not meeting grain requirements. Additional analysis of food groups by race/ethnicity in the current study indicated that approximately 95% NHB and Hispanics and 90% of Asians and NHW did not meet the required three servings of dairy per day. Approximately 60% NHB, 66% Asians, 85% Hispanics, and 66% NHW consumed less than three servings/day of the protein/meat group (5 and 5.5 are the requirements for women and men, respectively). Fruit and vegetable intake did not differ by race/ethnicity in the current study. 

Vitamin and mineral supplementation and exercise, associated with bone mass, were not measured in the current study. Vitamin D supplementation in bread improved physical function (locomotion and daily activities) over time in older adults living in a nursing home [21]. Another potential confounder of diet adequacy, physical function, and osteoporosis is exercise, which was not measured for this survey. Several randomized controlled trials of exercise for older adults with osteoporosis indicate improvements in physical function as compared to controls [22,23]. 

Bone mineral density was positively associated with greater total fruit and vegetable intake and lower rates of osteoporosis for Chinese adults aged 40–75 years with low body mass indices (<24 kg/m^2^) [24]. The association of increases in bone mass associated with increased intake of fruits and vegetables may not be valid in our primarily Caucasian population due, in part, to genetic and lifestyle differences. There is significant evidence that high-protein (found primarily in the meat and dairy groups) is necessary for elder’s bone health and muscle mass as long as the protein threshold is not exceeded for persons with inadequate kidney function [25]. According to the current study of congregate meal participants, the majority did not meet the requirements for dairy or meat.

Inadequate nutrition affects community-dwelling elders in the United States [20,26], as well as internationally [20,21]. Medical conditions, medications, physiological loss of taste, malabsorption, lack of resources for acquiring and preparing healthy foods, and social factors, such as loneliness and isolation, are among the factors contributing to inadequate nutrition in older adults [26]. Inadequate nutrition for older adults in Ireland was common, particularly lower than recommended intakes of vitamins and minerals needed for bone health: calcium magnesium, and vitamin D [27]. Over one-third (37.9%) reported consuming the recommended five or more servings of fruit and vegetables in an Iranian sample of adults, 65 years and older [28]; whereas, less than 25% consumed the recommended four servings of fruits and vegetables per day in our study.

In the present study, participants with osteoporosis were more-likely to have difficulty performing active daily living skills, a poorer quality of life, and a lower self-rated health as compared to participants without osteoporosis. Our results agreed with a study of community-dwelling adults aged 75 and older in Germany where osteoporosis negatively affected active daily living skills [29]; albeit, our study indicated an association, only. 

Finally, in the present study, several sex differences were found for osteoporosis and diet. Osteoporosis was more common in females as compared to males. Males consumed more servings of desserts and grains as compared to females; however, there were no differences in daily servings of meat, dairy, and fruits and vegetables. Our study agrees, in part, with Powers et al. [27], who reported that adult males had particularly poorer nutrition patterns as compared to females, consuming more calorie-dense, processed foods, in an Irish cohort of community-dwelling adults. 

This study has several limitations that should be mentioned. Causality cannot be assumed due cross-sectional design. Bone mass is determined largely by genetic factors; albeit, 10–50% of bone mass and structure is attributed to lifestyle factors such as diet, smoking, alcohol consumption, and physical activity [12]. Genetic factors, smoking, and alcohol consumption contribute to bone loss and were not assessed in this survey. Diet adequacy was based on the interviewers’ interpretation of participants’ reports and may be subject to recall bias. The number of desserts was a pseudo-measure of added sugars and did not take into account sugar-sweetened beverages and added sugars in other foods and beverages. Other factors associated with prevention of osteoporosis, dietary supplementation, particularly calcium, and vitamin D, and exercise were not measured in this survey. Although food insecurity was measured, the barrier of food cost and resources necessary for healthy eating may have played a role in inadequate food intake (reduced servings of major food groups). There were twice as many females as males; as such, the odds ratio for their differences may have been inflated. Although this study represents congregate meal site attendees across the United States, it cannot be generalized to community-dwelling older adult who do not participate in congregate meal programs.

## 5. Conclusions

People with osteoporosis had lower self-rated health and poorer physical health than people without osteoporosis. Even though congregate meal sites offer nutritious meals, the people surveyed were likely to have undernutrition. In particular they were found to have inadequate consumption of protein, dairy, and produce (a major vitamin and mineral source) essential for bone health despite. Strategies to increase participation in nutrition education may benefit health in this population.

## Figures and Tables

**Table 1:** Participants’ characteristics (*N* = 888).

Variable	Percent (95th CI)	Unweighted Numbers	*p*	p *
**Sociodemographic Factors**				
Age category (years)			<0.001	<0.001
60–64	5.4 (3.7, 7.8)	64		
65–74	40.2 (36.6, 44.1)	318		
75–84	35.5 (30.9, 40.3)	326		
85 and older	18.9 (15.6, 22.7)	180		
Sex			<0.001	<0.001
Male	34.3 (29.9, 39.0)	282		
Female	65.7 (62.0, 70.1)	606		
Race			<0.001	<0.001
Black	10.7 (7.6, 14.8)	138		
Asian/Am Indian/Pacific Islander	3.3 (1.8, 6.0)	31		
Hispanic	12.3 (8.5, 17.5)	41		
Non-Hispanic White	73.7 (67.9, 78.8)	669		
Education			0.002	0.002
Less than High School	16.8 (11.6, 23.7)	147		
HS or GED	33.1 (26.9, 40.0)	310		
Some College/Associate Degree	31.9 (26.7, 37.6)	271		
College degree or greater	18.1 (13.4, 24.1)	159		
Marital Status			0.007	ns
Currently married (yes)	39.8 (32.9, 47.1)	349		
Single, divorced, or widowed	60.2 (52.9, 67.1)	535		
Food Security			<0.001	<0.001
Enough money to buy food	84.6 (78.3, 89.3)	723		
Not enough money	15.4 (10.7, 21.7)	150		
Income level			0.138	ns
Below 20,000/year	45.1 (38.7, 51.6)	349		
at or above 20,000/year	54.9 (48.4, 61.3)	405		
**Health**				
Diabetes			<0.001	<0.001
yes	31.6 (27.1, 36.5)	277		
no	68.4 (63.5, 72.9)			
Physical health interferes with accomplishments			<0.001	<0.001
Rarely or not at all	25.0 (18.8, 32.4)	241		
Some of the time	27.8 (22.5, 33.9)	262		
All or most of the time	47.2 (41.9, 52.7)	374		
Osteoporosis			<0.001	<0.001
yes	20.1 (15.4, 25.7)	187		
no	79.9 (74.3, 84.6)	701		
Osteoporosis * sex			0.020	ns
Males (yes)	17.2 (8.7, 31.3)	20		
Females (yes)	82.8 (68.7, 91.3)	167		
**Services Attended**				
Nutrition education at site (yes)	11.7 (7.5,17.8)	102	<0.001	<0.001
Exercise at site (yes)	36.0 (30.1, 42.4)	315	<0.001	<0.001
Health screening at site (yes)	36.9 (29.6, 44.9)	339	<0.001	<0.001

Note. Frequencies were determined by Pearson chi square. Osteoporosis was considered affirmative if the participant recalled being told by a medical doctor. Health screenings are provided at all sites. Nutrition education and exercise sessions are not provided by all sites. *Abbreviations:* Am Indian is American Indian or Alaskan Native. Pacific Islander is Native Hawaiian or ‘other’ Pacific Islander. HS = high school. GED = General Equivalency Diploma. Osteoporosis * Sex is the level of osteoporosis for each sex, a test for modification of osteoporosis by sex. Testing 14 variable, the Bonferroni correction for an alpha of 95% would be *p* * < 0.003 and ns = not significant.

**Table 2:** Chi-square analysis of difficulties with physical function and percent osteoporosis.

Parameter	Osteoporosis	No Osteoporosis	Total	*p*	p *
Physical health interferes little or none with my accomplishments	31.9 (20.3, 46.2)	51.1 (45.7, 56.4)	47.2 (41.9, 52.7)	0.004	0.004
Health limits moderate activities a lot	31.5 (22.1, 42.7)	18.9 (14.2, 24.7)	21.3 (17.3, 26.0)	0.026	ns
Health limits climbing stairs a lot	32.2 (20.3, 46.9)	22.8 (17.1, 29.7.2)	24.7 (19.8, 30.2)	0.037	ns
Difficulty shopping or visiting doctors	27.4 (19.1, 37.7)	15.3 (10.8, 21.3)	17.7 (13.5, 23.0)	0.018	ns
Difficulty with light housework	17.8 (10.0, 29.7)	9.8 (6.8, 14.0)	11.4 (8.1, 16.0)	0.048	ns
Male	17.2 (8.7, 31.3)	38.6 (32.1, 45.5)	34.3 (29.9, 39.0)	0.020	ns
Female	82.8 (68.7, 91.3)	61.4 (54.5, 67.9)	65.7 (62.0, 70.1)	-	
	100% within sex	100% within osteoporosis	100% within osteoporosis		

Note. Data are percent (95th CI). There were no significant differences for osteoporosis status in race/ethnicity (*p* = 0.470), age-group (60–85 and over) (*p* = 0.569), education (*p* = 0.930), marital status (*p* = 0.736), food security (*p* = 0.353). Reporting receiving nutrition education, exercise, or health screenings on site did not differ by osteoporosis status. Osteoporosis was reported as recalling a medical doctor told them they had osteoporosis. Symbol, *p* * is with Bonferroni correction for testing 6 variables at an alpha of 95%. Only variables with *p* < 0.008 are considered significant and ns = not significant.

**Table 3:** Chi-squared test for the estimated proportion of calories consumed at the site and total servings per day (home and site, combined) of major food groups and dessert by sex.

Parameter	Males % (95th CI)	Females % (95th CI)	Total % (95th CI)	*p*	p *
Estimated 1/3 to 1/2 calories/day from Congregate Meals	51.5 (42.9, 59.9)	32.3 (24.0,41.9)	38.9 (33.2, 45.0)	0.001	0.001
Dairy				0.502	ns
3 or more servings/day (recommended)	14.0 (8.2, 22.9)	9.9 (6.9, 13.8)	11.3 (6.9, 13.8)		
at least 2 servings/day	25.2 (19.1, 34.1)	29.6 (21.4, 39.3)	28.1 (22.4, 34.5)		
0–1 servings/day	60.7 (53.0, 67.9)	60.6 (52.5, 69.0)	60.6 (53.5, 67.3)		
Total fruits and vegetables *				0.232	ns
5 or more servings/day	31.3 (23.3, 40.7)	34.6 (27.4, 42.5)	33.5 (27.7, 39.8)		
4 servings/day (recommendation 3.5, women and 4.5, men)	17.1 (10.6, 26.4)	24.3 (16.9, 33.5)	21.8 (16.1, 28.9)		
3 or fewer servings/day	51.6 (40.6, 62.4)	41.1 (30.3, 52.8)	44.7 (35.9, 53.9)		
Eat fruits and vegetables at site (yes)	95.7 (86.6, 98.7)	96.3 (94.2, 97.6)	96.1 (93.3, 97.7)	0.821	ns
Meat/protein				0.442	ns
5 or more servings/day (Recommended)	6.4 (3.3, 11.9)	3.8 (2.1, 6.9)	4.7 (3.0, 7.4)		
3-4 servings/day	28.2 (21.2, 33.2)	27.3 (21.4, 34.1)	27.1 (22.4, 32.3)		
1–2 servings/day	66.8 (60.6, 72.5)	68.9 (60.9, 75.9)	68.2 (62.3, 73.5)		
Grain				0.006	0.006
5 or more servings/day Recommendation 8 and 9 servings/day (women and men, respectively)	14.8 (9.3, 22.7)	9.0 (6.4, 12.4)	11.0 (8.0, 15.1)		
3–4 servings/day	40.0 (33.1, 47.3)	31.9 (27.2, 37.0)	34.7 (30.8, 38.9)		
1–2 servings/day	45.2 (36.8, 53.8)	59.2 (53.6, 64.5)	54.3 (48.9, 59.5)		
Dessert				0.018	ns
none	17.3 (11.0, 26.2)	23.7 (18.3, 30.0)	21.5 (17.8, 25.7)		
1 serving/day	51.3 (39.3, 63.2)	60.8 (53.8, 67.4)	57.6 (51.3, 63.6)		
2 or more servings/day **	31.3 (23.7, 40.2)	15.5 (11.6, 20.6)	20.9 (17.2, 25.2)		

Note. The proportion of calories were compared by average total servings per day at home and at the site. Data were provided in whole servings, only Meat/protein includes beans, seeds, tofu, fish, eggs, chicken, and red meats. Grain includes bread and cooked grains. * Total fruits and vegetables do not include potatoes, since they are not considered in this group, nutritionally. ** Recommendations for added sugars are no more than 10% of calories; two or more servings/day of desserts per day is likely to exceed this recommendation for older adults who are consuming less than the recommended amount from all dietary groups. Recommendations are based on the nearest whole serving size for adults >50 years of age [11]. The column *p* * was considered (<0.007) after the Bonferroni correction for seven variables was applied to 95% alpha and ns = not significant.

**Table 4:** Logistic regression model for participant characteristics and odds of osteoporosis.

Variables	B coefficients (95th CI)	df	OR (95th CI)	*p*	p *
Intercept	−1.30 (−2.27, −0.32)	1, 64	0.27 (0.10, 0.73)	0.010	ns
Sex—female	1.20 (0.35, 2.05)	1, 64	3.32 (1.42, 7.77)	0.007	0.007
Male (reference)	0		1.00		
Age category (years)		3, 62	ns	0.565	ns
60–64	−0.77 (−1.97, 0.43)	1, 64	0.46 (0.14, 1.54)	0.205	
65–74	−0.30 (−1.33, 0.74)	1, 64	0.74 (0.26, 2.09)	0.568	
75–84	−0.45 (−0.13, 0.39)	1, 64	0.63 (0.27, 1.48)	0.283	
85 and above (reference)	0		1.00		
Race/ethnicity		3, 62	ns	0.075	ns
Black	1.41 (−2.47, −0.34)	1, 64	0.24 (0.08, 0.71)	0.010	
Asian/Pacific Islander	0.29 (−0.75, 1.35)	1, 64	1.34 (0.47, 3.85)	0.576	
Hispanic	0.04 (−1.94, 2.01)	1, 64	1.04 (0.14, 7.47	0.971	
White, non-Hispanic (reference)	0		1.00		
Food Security (Yes—enough to buy food)	0.44 (−0.29, 1.17)	1, 64	1.56 (0.75, 3.22)	0.229	ns
Self-Rated Health		2, 63		0.030	ns
Fair to poor	0.80 (0.15, 1.46)		2.24 (1.16, 4.30)	0.017	
Good	0.10 (−0.54, 0.75)		1.11 (0.58, 2.11)	0.750	
very good to excellent (reference)	0		1.00	-	

Note. This was the most parsimonious model of risk-factors for osteoporosis and SRH. Marriage, education, poverty, and diabetes achieved a fitted model (significant, corrected model), but did not contribute to the model (goodness of fit). df = degrees of freedom. Variable with ‘ns’ are not significant and the corresponding odds ratios of their parameters are, therefore considered insignificant. Asian category is American Indian or Alaskan Native. Pacific Islander is Native Hawaiian or ‘other’ Pacific Islander. Column *p* * is the Bonferroni correction for 5 variables and an alpha of 95% is *p* * < 0.010 and ns = not significant.
